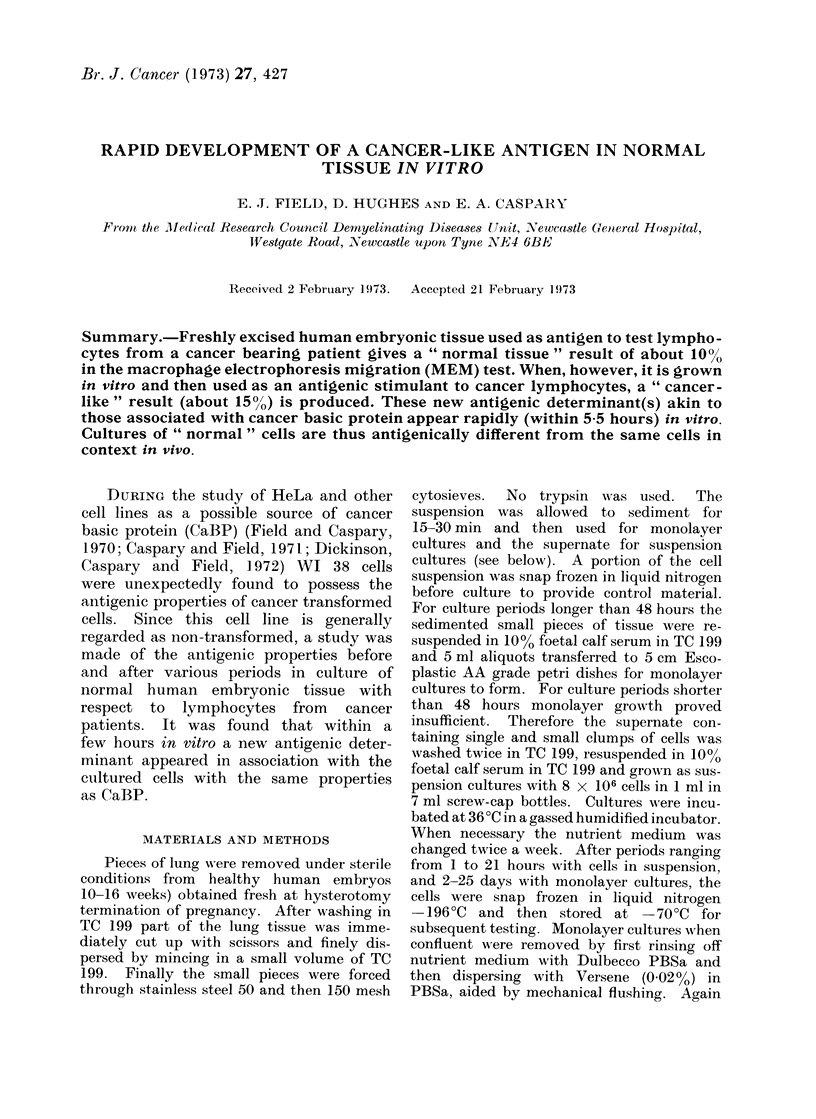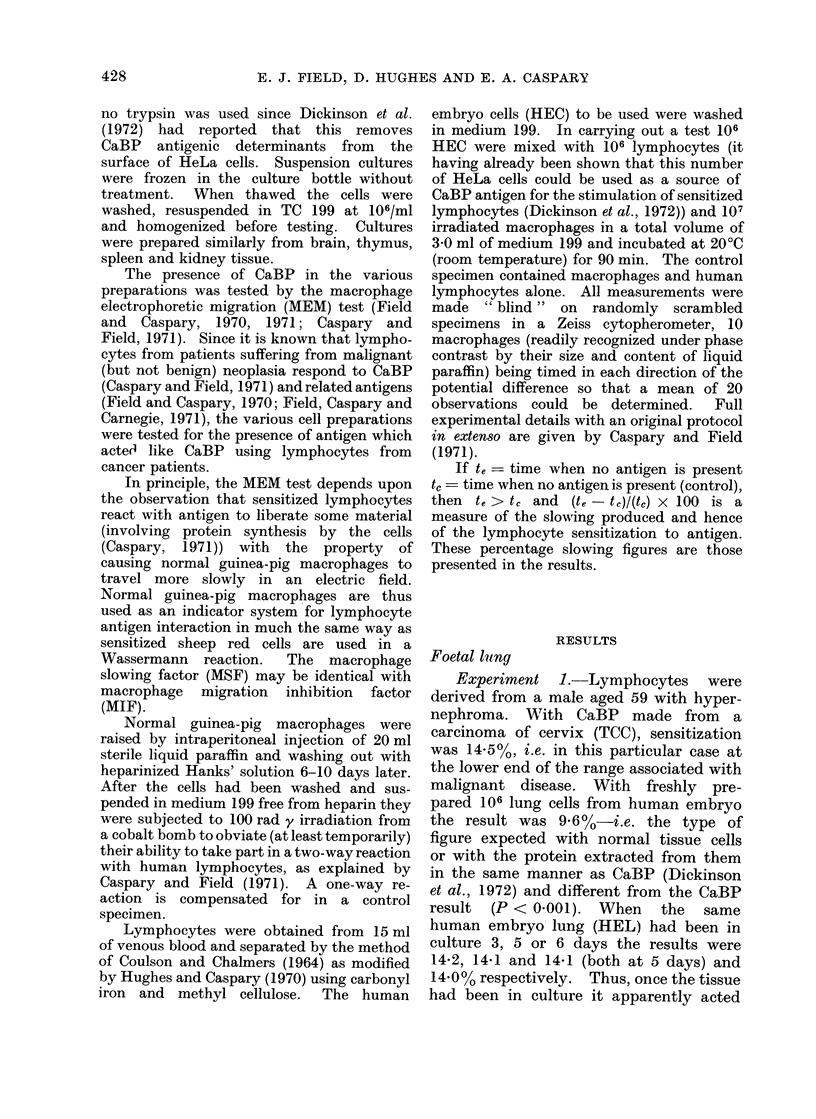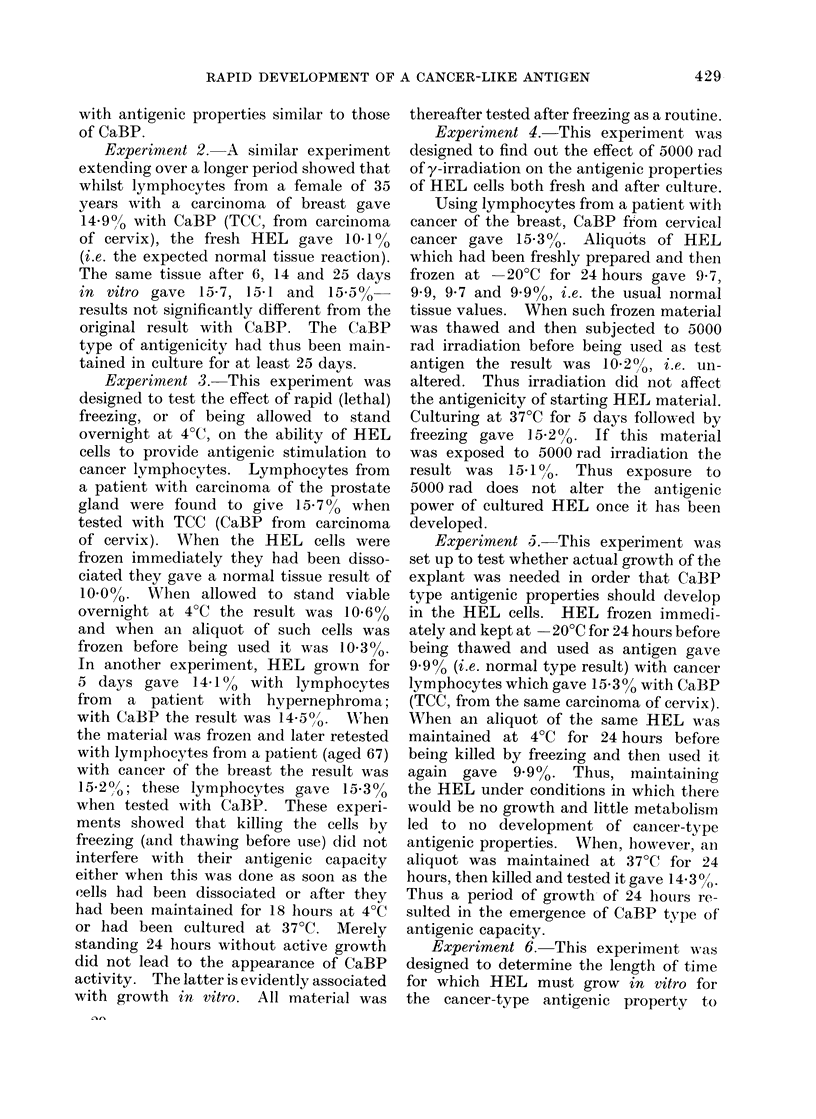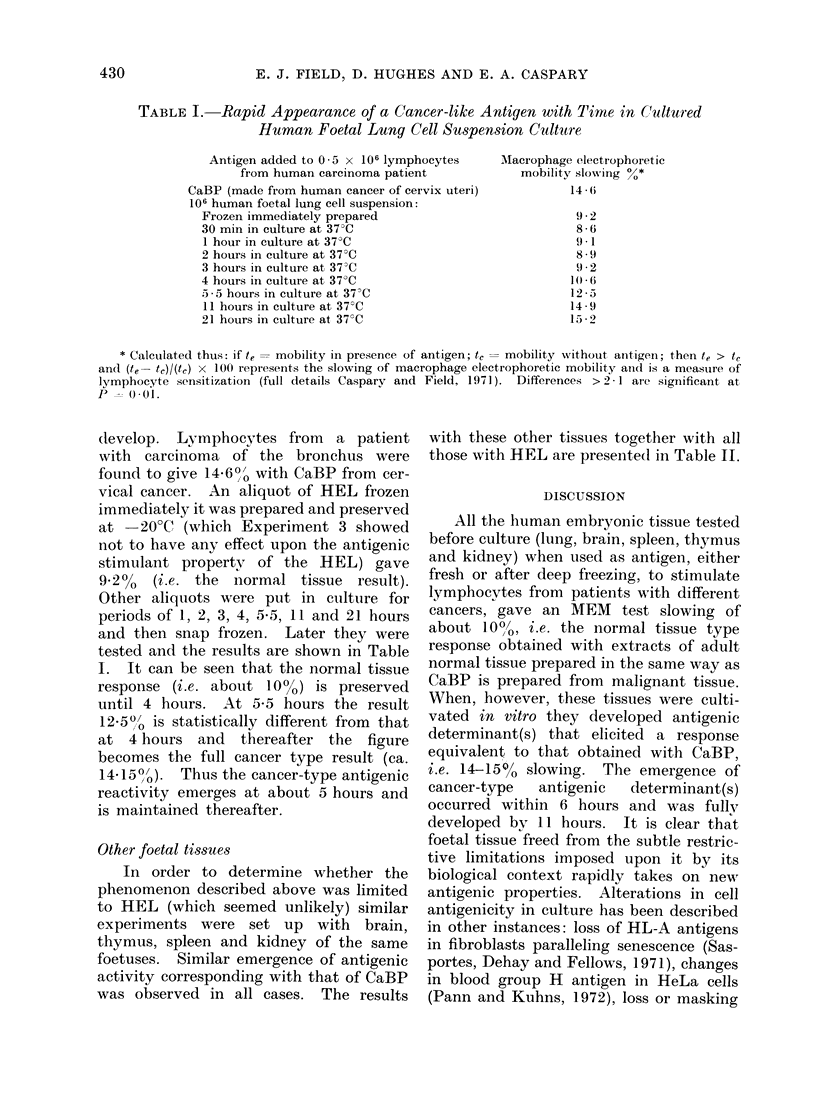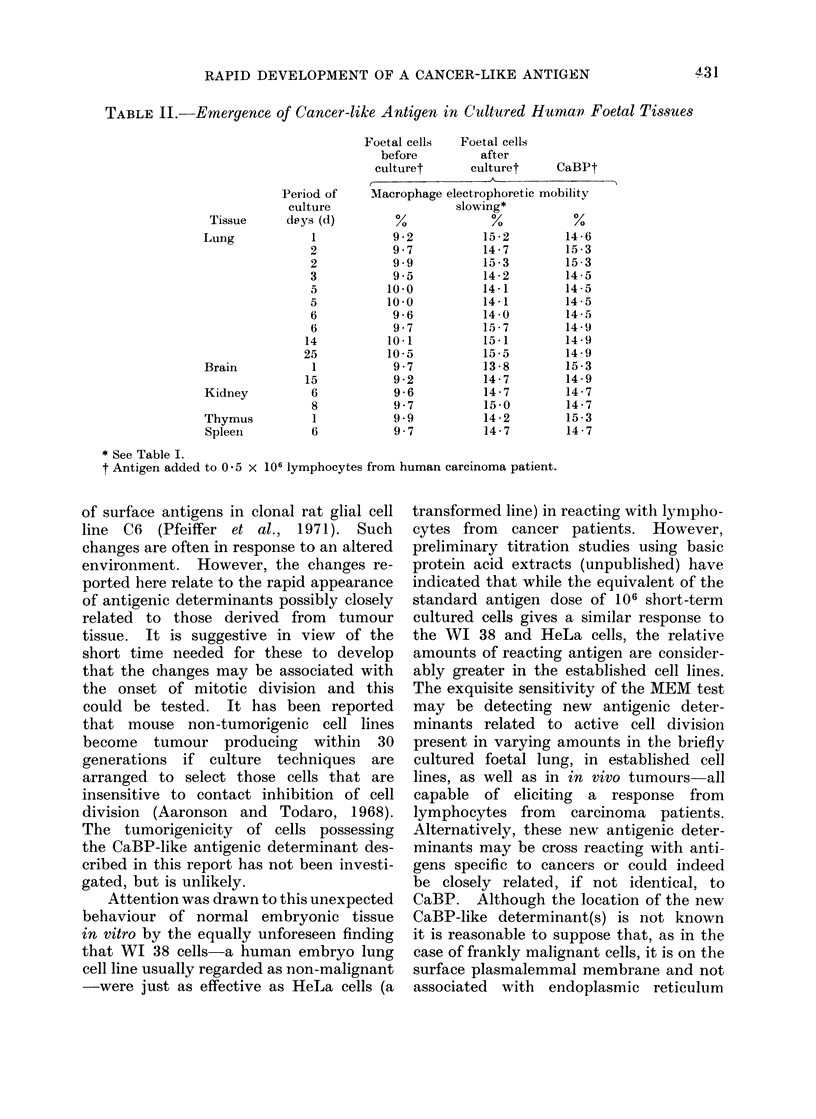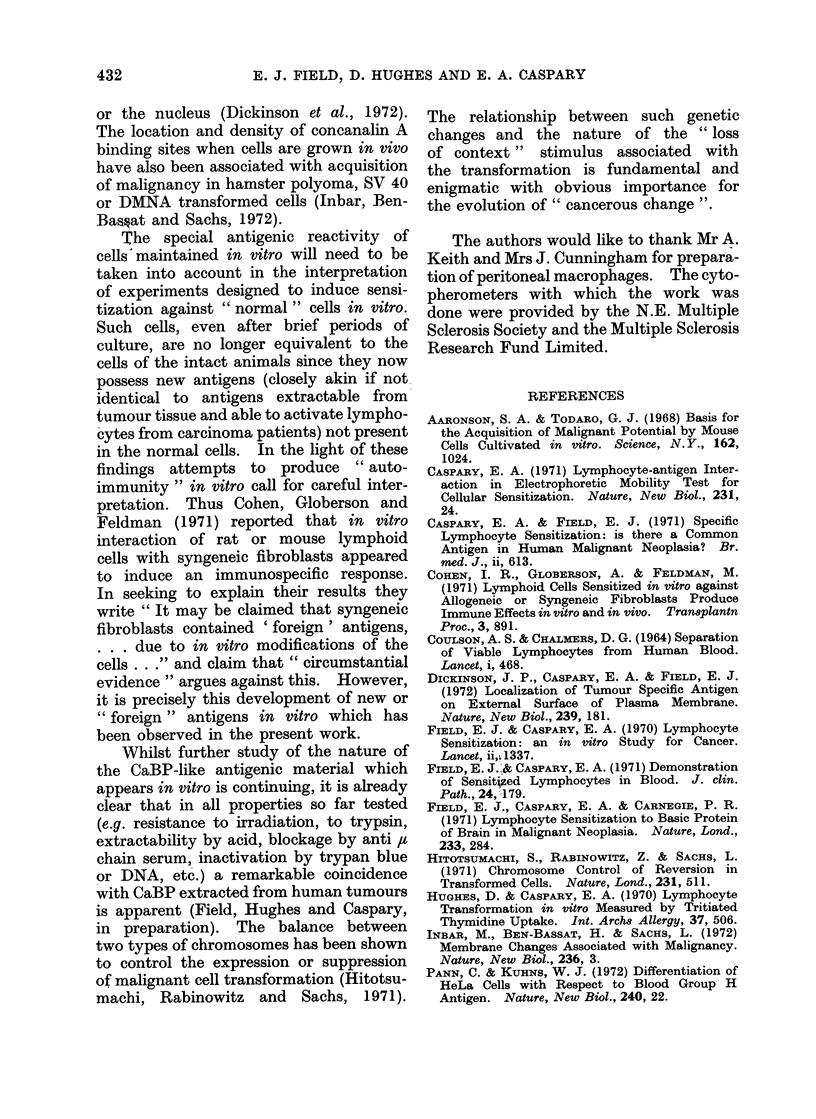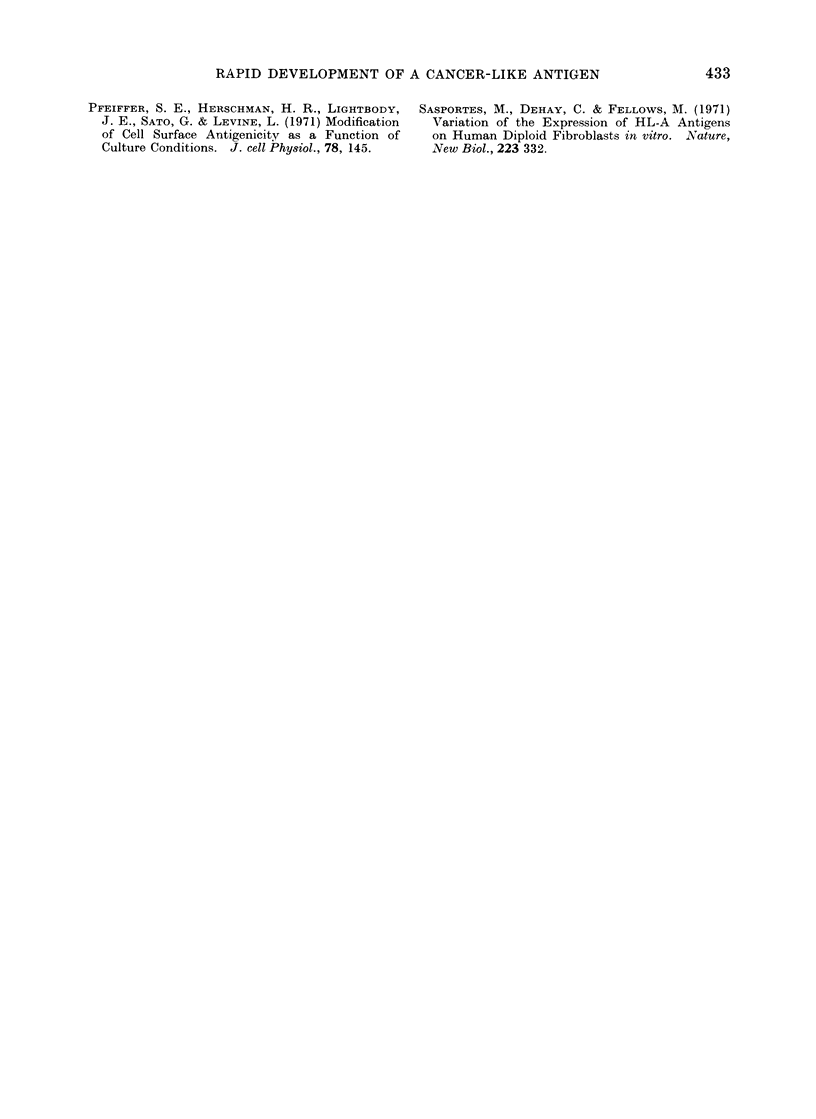# Rapid Development of a Cancer-like Antigen in Normal Tissue in vitro

**DOI:** 10.1038/bjc.1973.53

**Published:** 1973-06

**Authors:** E. J. Field, D. Hughes, E. A. Caspary

## Abstract

Freshly excised human embryonic tissue used as antigen to test lymphocytes from a cancer bearing patient gives a “normal tissue” result of about 10% in the macrophage electrophoresis migration (MEM) test. When, however, it is grown *in vitro* and then used as an antigenic stimulant to cancer lymphocytes, a “cancerlike” result (about 15%) is produced. These new antigenic determinant(s) akin to those associated with cancer basic protein appear rapidly (within 5.5 hours) *in vitro*. Cultures of “normal” cells are thus antigenically different from the same cells in context *in vivo*.


Br. J. Cancer (1973) 27, 427

### RAPID DEVELOPMENT OF A CANCER-LIKE ANTIGEN IN NORMAL TISSUE *IN VITRO*

E. J. FIELD, D. HUGHES AND E. A. CASPARY

*From the Medical Research Council Demyelinating Diseases Unit, Newcastle General Hospital, Westgate Road, Newcastle upon Tyne NE4 6BE*

Received 2 February 1973. Accepted 21 February 1973

**Summary.—Freshly excised human embryonic tissue used as antigen to test lymphocytes from a cancer bearing patient gives a " normal tissue " result of about 10% in the macrophage electrophoresis migration (MEM) test. When, however, it is grown *in vitro* and then used as an antigenic stimulant to cancer lymphocytes, a " cancer-like " result (about 15%) is produced. These new antigenic determinant(s) akin to those associated with cancer basic protein appear rapidly (within 5·5 hours) *in vitro*. Cultures of " normal " cells are thus antigenically different from the same cells in context *in vivo*.**

DURING the study of HeLa and other cell lines as a possible source of cancer basic protein (CaBP) (Field and Caspary, 1970; Caspary and Field, 1971; Dickinson, Caspary and Field, 1972) WI 38 cells were unexpectedly found to possess the antigenic properties of cancer transformed cells. Since this cell line is generally regarded as non-transformed, a study was made of the antigenic properties before and after various periods in culture of normal human embryonic tissue with respect to lymphocytes from cancer patients. It was found that within a few hours *in vitro* a new antigenic determinant appeared in association with the cultured cells with the same properties as CaBP.

### MATERIALS AND METHODS

Pieces of lung were removed under sterile conditions from healthy human embryos 10–16 weeks) obtained fresh at hysterotomy termination of pregnancy. After washing in TC 199 part of the lung tissue was immediately cut up with scissors and finely dispersed by mincing in a small volume of TC 199. Finally the small pieces were forced through stainless steel 50 and then 150 mesh cytosieves. No trypsin was used. The suspension was allowed to sediment for 15–30 min and then used for monolayer cultures and the supernate for suspension cultures (see below). A portion of the cell suspension was snap frozen in liquid nitrogen before culture to provide control material. For culture periods longer than 48 hours the sedimented small pieces of tissue were resuspended in 10% foetal calf serum in TC 199 and 5 ml aliquots transferred to 5 cm Esco-plastic AA grade petri dishes for monolayer cultures to form. For culture periods shorter than 48 hours monolayer growth proved insufficient. Therefore the supernate containing single and small clumps of cells was washed twice in TC 199, resuspended in 10% foetal calf serum in TC 199 and grown as suspension cultures with $8 \times 10^6$ cells in 1 ml in 7 ml screw-cap bottles. Cultures were incubated at 36 °C in a gassed humidified incubator. When necessary the nutrient medium was changed twice a week. After periods ranging from 1 to 21 hours with cells in suspension, and 2–25 days with monolayer cultures, the cells were snap frozen in liquid nitrogen −196°C and then stored at −70°C for subsequent testing. Monolayer cultures when confluent were removed by first rinsing off nutrient medium with Dulbecco PBSa and then dispersing with Versene (0·02%) in PBSa, aided by mechanical flushing. Again no trypsin was used since Dickinson *et al.* (1972) had reported that this removes CaBP antigenic determinants from the surface of HeLa cells. Suspension cultures were frozen in the culture bottle without treatment. When thawed the cells were washed, resuspended in TC 199 at $10^6$/ml and homogenized before testing. Cultures were prepared similarly from brain, thymus, spleen and kidney tissue.

The presence of CaBP in the various preparations was tested by the macrophage electrophoretic migration (MEM) test (Field and Caspary, 1970, 1971; Caspary and Field, 1971). Since it is known that lymphocytes from patients suffering from malignant (but not benign) neoplasia respond to CaBP (Caspary and Field, 1971) and related antigens (Field and Caspary, 1970; Field, Caspary and Carnegie, 1971), the various cell preparations were tested for the presence of antigen which acted like CaBP using lymphocytes from cancer patients.

In principle, the MEM test depends upon the observation that sensitized lymphocytes react with antigen to liberate some material (involving protein synthesis by the cells (Caspary, 1971)) with the property of causing normal guinea-pig macrophages to travel more slowly in an electric field. Normal guinea-pig macrophages are thus used as an indicator system for lymphocyte antigen interaction in much the same way as sensitized sheep red cells are used in a Wassermann reaction. The macrophage slowing factor (MSF) may be identical with macrophage migration inhibition factor (MIF).

Normal guinea-pig macrophages were raised by intraperitoneal injection of 20 ml sterile liquid paraffin and washing out with heparinized Hanks' solution 6–10 days later. After the cells had been washed and suspended in medium 199 free from heparin they were subjected to 100 rad $\gamma$ irradiation from a cobalt bomb to obviate (at least temporarily) their ability to take part in a two-way reaction with human lymphocytes, as explained by Caspary and Field (1971). A one-way reaction is compensated for in a control specimen.

Lymphocytes were obtained from 15 ml of venous blood and separated by the method of Coulson and Chalmers (1964) as modified by Hughes and Caspary (1970) using carbonyl iron and methyl cellulose. The human embryo cells (HEC) to be used were washed in medium 199. In carrying out a test $10^6$ HEC were mixed with $10^6$ lymphocytes (it having already been shown that this number of HeLa cells could be used as a source of CaBP antigen for the stimulation of sensitized lymphocytes (Dickinson *et al.*, 1972)) and $10^7$ irradiated macrophages in a total volume of 3·0 ml of medium 199 and incubated at 20°C (room temperature) for 90 min. The control specimen contained macrophages and human lymphocytes alone. All measurements were made "blind" on randomly scrambled specimens in a Zeiss cytopherometer, 10 macrophages (readily recognized under phase contrast by their size and content of liquid paraffin) being timed in each direction of the potential difference so that a mean of 20 observations could be determined. Full experimental details with an original protocol *in extenso* are given by Caspary and Field (1971).

If $t_e$ = time when no antigen is present $t_c$ = time when no antigen is present (control), then $t_e > t_c$ and $(t_e - t_c)/(t_c) \times 100$ is a measure of the slowing produced and hence of the lymphocyte sensitization to antigen. These percentage slowing figures are those presented in the results.

### RESULTS

### *Foetal lung*

*Experiment 1.*—Lymphocytes were derived from a male aged 59 with hypernephroma. With CaBP made from a carcinoma of cervix (TCC), sensitization was 14·5%, *i.e.* in this particular case at the lower end of the range associated with malignant disease. With freshly prepared $10^6$ lung cells from human embryo the result was 9·6%—*i.e.* the type of figure expected with normal tissue cells or with the protein extracted from them in the same manner as CaBP (Dickinson *et al.*, 1972) and different from the CaBP result ($P < 0{\cdot}001$). When the same human embryo lung (HEL) had been in culture 3, 5 or 6 days the results were 14·2, 14·1 and 14·1 (both at 5 days) and 14·0% respectively. Thus, once the tissue had been in culture it apparently acted with antigenic properties similar to those of CaBP.

*Experiment 2.*—A similar experiment extending over a longer period showed that whilst lymphocytes from a female of 35 years with a carcinoma of breast gave 14·9% with CaBP (TCC, from carcinoma of cervix), the fresh HEL gave 10·1% (*i.e.* the expected normal tissue reaction). The same tissue after 6, 14 and 25 days *in vitro* gave 15·7, 15·1 and 15·5%—results not significantly different from the original result with CaBP. The CaBP type of antigenicity had thus been maintained in culture for at least 25 days.

*Experiment 3.*—This experiment was designed to test the effect of rapid (lethal) freezing, or of being allowed to stand overnight at 4°C, on the ability of HEL cells to provide antigenic stimulation to cancer lymphocytes. Lymphocytes from a patient with carcinoma of the prostate gland were found to give 15·7% when tested with TCC (CaBP from carcinoma of cervix). When the HEL cells were frozen immediately they had been dissociated they gave a normal tissue result of 10·0%. When allowed to stand viable overnight at 4°C the result was 10·6% and when an aliquot of such cells was frozen before being used it was 10·3%. In another experiment, HEL grown for 5 days gave 14·1% with lymphocytes from a patient with hypernephroma; with CaBP the result was 14·5%. When the material was frozen and later retested with lymphocytes from a patient (aged 67) with cancer of the breast the result was 15·2%; these lymphocytes gave 15·3% when tested with CaBP. These experiments showed that killing the cells by freezing (and thawing before use) did not interfere with their antigenic capacity either when this was done as soon as the cells had been dissociated or after they had been maintained for 18 hours at 4°C or had been cultured at 37°C. Merely standing 24 hours without active growth did not lead to the appearance of CaBP activity. The latter is evidently associated with growth *in vitro*. All material was thereafter tested after freezing as a routine.

*Experiment 4.*—This experiment was designed to find out the effect of 5000 rad of $\gamma$-irradiation on the antigenic properties of HEL cells both fresh and after culture.

Using lymphocytes from a patient with cancer of the breast, CaBP from cervical cancer gave 15·3%. Aliquots of HEL which had been freshly prepared and then frozen at −20°C for 24 hours gave 9·7, 9·9, 9·7 and 9·9%, *i.e.* the usual normal tissue values. When such frozen material was thawed and then subjected to 5000 rad irradiation before being used as test antigen the result was 10·2%, *i.e.* unaltered. Thus irradiation did not affect the antigenicity of starting HEL material. Culturing at 37°C for 5 days followed by freezing gave 15·2%. If this material was exposed to 5000 rad irradiation the result was 15·1%. Thus exposure to 5000 rad does not alter the antigenic power of cultured HEL once it has been developed.

*Experiment 5.*—This experiment was set up to test whether actual growth of the explant was needed in order that CaBP type antigenic properties should develop in the HEL cells. HEL frozen immediately and kept at −20°C for 24 hours before being thawed and used as antigen gave 9·9% (*i.e.* normal type result) with cancer lymphocytes which gave 15·3% with CaBP (TCC, from the same carcinoma of cervix). When an aliquot of the same HEL was maintained at 4°C for 24 hours before being killed by freezing and then used it again gave 9·9%. Thus, maintaining the HEL under conditions in which there would be no growth and little metabolism led to no development of cancer-type antigenic properties. When, however, an aliquot was maintained at 37°C for 24 hours, then killed and tested it gave 14·3%. Thus a period of growth of 24 hours resulted in the emergence of CaBP type of antigenic capacity.

*Experiment 6.*—This experiment was designed to determine the length of time for which HEL must grow *in vitro* for the cancer-type antigenic property to

TABLE I.—*Rapid Appearance of a Cancer-like Antigen with Time in Cultured Human Foetal Lung Cell Suspension Culture*

| Antigen added to $0 \cdot 5 \times 10^6$ lymphocytes from human carcinoma patient | Macrophage electrophoretic mobility slowing %* |
|---|---|
| CaBP (made from human cancer of cervix uteri) | 14·6 |
| $10^6$ human foetal lung cell suspension: | |
| Frozen immediately prepared | 9·2 |
| 30 min in culture at 37°C | 8·6 |
| 1 hour in culture at 37°C | 9·1 |
| 2 hours in culture at 37°C | 8·9 |
| 3 hours in culture at 37°C | 9·2 |
| 4 hours in culture at 37°C | 10·6 |
| 5·5 hours in culture at 37°C | 12·5 |
| 11 hours in culture at 37°C | 14·9 |
| 21 hours in culture at 37°C | 15·2 |

* Calculated thus: if $t_e$ = mobility in presence of antigen; $t_c$ = mobility without antigen; then $t_e > t_c$ and $(t_e - t_c)/(t_c) \times 100$ represents the slowing of macrophage electrophoretic mobility and is a measure of lymphocyte sensitization (full details Caspary and Field, 1971). Differences >2·1 are significant at $P = 0 \cdot 01$.

develop. Lymphocytes from a patient with carcinoma of the bronchus were found to give 14·6% with CaBP from cervical cancer. An aliquot of HEL frozen immediately it was prepared and preserved at −20°C (which Experiment 3 showed not to have any effect upon the antigenic stimulant property of the HEL) gave 9·2% (*i.e.* the normal tissue result). Other aliquots were put in culture for periods of 1, 2, 3, 4, 5·5, 11 and 21 hours and then snap frozen. Later they were tested and the results are shown in Table I. It can be seen that the normal tissue response (*i.e.* about 10%) is preserved until 4 hours. At 5·5 hours the result 12·5% is statistically different from that at 4 hours and thereafter the figure becomes the full cancer type result (ca. 14·15%). Thus the cancer-type antigenic reactivity emerges at about 5 hours and is maintained thereafter.

*Other foetal tissues*

In order to determine whether the phenomenon described above was limited to HEL (which seemed unlikely) similar experiments were set up with brain, thymus, spleen and kidney of the same foetuses. Similar emergence of antigenic activity corresponding with that of CaBP was observed in all cases. The results with these other tissues together with all those with HEL are presented in Table II.

### DISCUSSION

All the human embryonic tissue tested before culture (lung, brain, spleen, thymus and kidney) when used as antigen, either fresh or after deep freezing, to stimulate lymphocytes from patients with different cancers, gave an MEM test slowing of about 10%, *i.e.* the normal tissue type response obtained with extracts of adult normal tissue prepared in the same way as CaBP is prepared from malignant tissue. When, however, these tissues were cultivated *in vitro* they developed antigenic determinant(s) that elicited a response equivalent to that obtained with CaBP, *i.e.* 14–15% slowing. The emergence of cancer-type antigenic determinant(s) occurred within 6 hours and was fully developed by 11 hours. It is clear that foetal tissue freed from the subtle restrictive limitations imposed upon it by its biological context rapidly takes on new antigenic properties. Alterations in cell antigenicity in culture has been described in other instances: loss of HL-A antigens in fibroblasts paralleling senescence (Sasportes, Dehay and Fellows, 1971), changes in blood group H antigen in HeLa cells (Pann and Kuhns, 1972), loss or masking

TABLE II.—*Emergence of Cancer-like Antigen in Cultured Human Foetal Tissues*

| Tissue | Period of culture days (d) | Foetal cells before culture† | Foetal cells after culture† | CaBP† |
|---|---|---|---|---|
| | | Macrophage electrophoretic mobility slowing* | | |
| | | % | % | % |
| Lung | 1 | 9·2 | 15·2 | 14·6 |
| | 2 | 9·7 | 14·7 | 15·3 |
| | 2 | 9·9 | 15·3 | 15·3 |
| | 3 | 9·5 | 14·2 | 14·5 |
| | 5 | 10·0 | 14·1 | 14·5 |
| | 5 | 10·0 | 14·1 | 14·5 |
| | 6 | 9·6 | 14·0 | 14·5 |
| | 6 | 9·7 | 15·7 | 14·9 |
| | 14 | 10·1 | 15·1 | 14·9 |
| | 25 | 10·5 | 15·5 | 14·9 |
| Brain | 1 | 9·7 | 13·8 | 15·3 |
| | 15 | 9·2 | 14·7 | 14·9 |
| Kidney | 6 | 9·6 | 14·7 | 14·7 |
| | 8 | 9·7 | 15·0 | 14·7 |
| Thymus | 1 | 9·9 | 14·2 | 15·3 |
| Spleen | 6 | 9·7 | 14·7 | 14·7 |

* See Table I.

† Antigen added to $0 \cdot 5 \times 10^6$ lymphocytes from human carcinoma patient.

of surface antigens in clonal rat glial cell line C6 (Pfeiffer *et al.*, 1971). Such changes are often in response to an altered environment. However, the changes reported here relate to the rapid appearance of antigenic determinants possibly closely related to those derived from tumour tissue. It is suggestive in view of the short time needed for these to develop that the changes may be associated with the onset of mitotic division and this could be tested. It has been reported that mouse non-tumorigenic cell lines become tumour producing within 30 generations if culture techniques are arranged to select those cells that are insensitive to contact inhibition of cell division (Aaronson and Todaro, 1968). The tumorigenicity of cells possessing the CaBP-like antigenic determinant described in this report has not been investigated, but is unlikely.

Attention was drawn to this unexpected behaviour of normal embryonic tissue *in vitro* by the equally unforeseen finding that WI 38 cells—a human embryo lung cell line usually regarded as non-malignant —were just as effective as HeLa cells (a transformed line) in reacting with lymphocytes from cancer patients. However, preliminary titration studies using basic protein acid extracts (unpublished) have indicated that while the equivalent of the standard antigen dose of $10^6$ short-term cultured cells gives a similar response to the WI 38 and HeLa cells, the relative amounts of reacting antigen are considerably greater in the established cell lines. The exquisite sensitivity of the MEM test may be detecting new antigenic determinants related to active cell division present in varying amounts in the briefly cultured foetal lung, in established cell lines, as well as in *in vivo* tumours—all capable of eliciting a response from lymphocytes from carcinoma patients. Alternatively, these new antigenic determinants may be cross reacting with antigens specific to cancers or could indeed be closely related, if not identical, to CaBP. Although the location of the new CaBP-like determinant(s) is not known it is reasonable to suppose that, as in the case of frankly malignant cells, it is on the surface plasmalemmal membrane and not associated with endoplasmic reticulum or the nucleus (Dickinson *et al.*, 1972). The location and density of concanalin A binding sites when cells are grown *in vivo* have also been associated with acquisition of malignancy in hamster polyoma, SV 40 or DMNA transformed cells (Inbar, Ben-Bassat and Sachs, 1972).

The special antigenic reactivity of cells maintained *in vitro* will need to be taken into account in the interpretation of experiments designed to induce sensitization against " normal " cells *in vitro*. Such cells, even after brief periods of culture, are no longer equivalent to the cells of the intact animals since they now possess new antigens (closely akin if not identical to antigens extractable from tumour tissue and able to activate lymphocytes from carcinoma patients) not present in the normal cells. In the light of these findings attempts to produce " autoimmunity " *in vitro* call for careful interpretation. Thus Cohen, Globerson and Feldman (1971) reported that *in vitro* interaction of rat or mouse lymphoid cells with syngeneic fibroblasts appeared to induce an immunospecific response. In seeking to explain their results they write " It may be claimed that syngeneic fibroblasts contained ' foreign ' antigens, . . . due to *in vitro* modifications of the cells . . ." and claim that " circumstantial evidence " argues against this. However, it is precisely this development of new or " foreign " antigens *in vitro* which has been observed in the present work.

Whilst further study of the nature of the CaBP-like antigenic material which appears *in vitro* is continuing, it is already clear that in all properties so far tested (*e.g.* resistance to irradiation, to trypsin, extractability by acid, blockage by anti $\mu$ chain serum, inactivation by trypan blue or DNA, etc.) a remarkable coincidence with CaBP extracted from human tumours is apparent (Field, Hughes and Caspary, in preparation). The balance between two types of chromosomes has been shown to control the expression or suppression of malignant cell transformation (Hitotsumachi, Rabinowitz and Sachs, 1971). The relationship between such genetic changes and the nature of the " loss of context " stimulus associated with the transformation is fundamental and enigmatic with obvious importance for the evolution of " cancerous change ".

The authors would like to thank Mr A. Keith and Mrs J. Cunningham for preparation of peritoneal macrophages. The cytopherometers with which the work was done were provided by the N.E. Multiple Sclerosis Society and the Multiple Sclerosis Research Fund Limited.

### REFERENCES

AARONSON, S. A. & TODARO, G. J. (1968) Basis for the Acquisition of Malignant Potential by Mouse Cells Cultivated *in vitro*. *Science, N.Y.*, **162**, 1024.

CASPARY, E. A. (1971) Lymphocyte-antigen Interaction in Electrophoretic Mobility Test for Cellular Sensitization. *Nature, New Biol.*, **231**, 24.

CASPARY, E. A. & FIELD, E. J. (1971) Specific Lymphocyte Sensitization: is there a Common Antigen in Human Malignant Neoplasia? *Br. med. J.*, ii, 613.

COHEN, I. R., GLOBERSON, A. & FELDMAN, M. (1971) Lymphoid Cells Sensitized *in vitro* against Allogeneic or Syngeneic Fibroblasts Produce Immune Effects *in vitro* and *in vivo*. *Transplantn Proc.*, **3**, 891.

COULSON, A. S. & CHALMERS, D. G. (1964) Separation of Viable Lymphocytes from Human Blood. *Lancet*, i, 468.

DICKINSON, J. P., CASPARY, E. A. & FIELD, E. J. (1972) Localization of Tumour Specific Antigen on External Surface of Plasma Membrane. *Nature, New Biol.*, **239**, 181.

FIELD, E. J. & CASPARY, E. A. (1970) Lymphocyte Sensitization: an *in vitro* Study for Cancer. *Lancet*, ii, 1337.

FIELD, E. J. & CASPARY, E. A. (1971) Demonstration of Sensitized Lymphocytes in Blood. *J. clin. Path.*, **24**, 179.

FIELD, E. J., CASPARY, E. A. & CARNEGIE, P. R. (1971) Lymphocyte Sensitization to Basic Protein of Brain in Malignant Neoplasia. *Nature, Lond.*, **233**, 284.

HITOTSUMACHI, S., RABINOWITZ, Z. & SACHS, L. (1971) Chromosome Control of Reversion in Transformed Cells. *Nature, Lond.*, **231**, 511.

HUGHES, D. & CASPARY, E. A. (1970) Lymphocyte Transformation *in vitro* Measured by Tritiated Thymidine Uptake. *Int. Archs Allergy*, **37**, 506.

INBAR, M., BEN-BASSAT, H. & SACHS, L. (1972) Membrane Changes Associated with Malignancy. *Nature, New Biol.*, **236**, 3.

PANN, C. & KUHNS, W. J. (1972) Differentiation of HeLa Cells with Respect to Blood Group H Antigen. *Nature, New Biol.*, **240**, 22.

PFEIFFER, S. E., HERSCHMAN, H. R., LIGHTBODY, J. E., SATO, G. & LEVINE, L. (1971) Modification of Cell Surface Antigenicity as a Function of Culture Conditions. *J. cell Physiol.*, **78**, 145.

SASPORTES, M., DEHAY, C. & FELLOWS, M. (1971) Variation of the Expression of HL-A Antigens on Human Diploid Fibroblasts *in vitro*. *Nature, New Biol.*, **223** 332.